# Using linked administrative and disease-specific databases to study end-of-life care on a population level

**DOI:** 10.1186/s12904-016-0159-7

**Published:** 2016-10-18

**Authors:** Arno Maetens, Robrecht De Schreye, Kristof Faes, Dirk Houttekier, Luc Deliens, Birgit Gielen, Cindy De Gendt, Patrick Lusyne, Lieven Annemans, Joachim Cohen

**Affiliations:** 1 End of Life Care Research Group, Vrije Universiteit Brussel (VUB), Brussels, Belgium & Ghent University, Ghent, Belgium; 2 Interuniversity Centre for Health Economics Research (I-CHER), Ghent University, Ghent, Belgium; 3 Department of medical oncology, Ghent University Hospital, Ghent, Belgium; 4 InterMutualistic Agency, Brussels, Belgium; 5 Belgian Cancer Registry, Brussels, Belgium; 6 Statistics Belgium, Brussels, Belgium

**Keywords:** End-of-life, Data linkage, Administrative databases, Disease-specific databases, Full-population

## Abstract

**Background:**

The use of full-population databases is under-explored to study the use, quality and costs of end-of-life care. Using the case of Belgium, we explored: (1) which full-population databases provide valid information about end-of-life care, (2) what procedures are there to use these databases, and (3) what is needed to integrate separate databases.

**Methods:**

Technical and privacy-related aspects of linking and accessing Belgian administrative databases and disease registries were assessed in cooperation with the database administrators and privacy commission bodies. For all relevant databases, we followed procedures in cooperation with database administrators to link the databases and to access the data.

**Results:**

We identified several databases as fitting for end-of-life care research in Belgium: the InterMutualistic Agency's national registry of health care claims data, the Belgian Cancer Registry including data on incidence of cancer, and databases administrated by Statistics Belgium including data from the death certificate database, the socio-economic survey and fiscal data. To obtain access to the data, approval was required from all database administrators, supervisory bodies and two separate national privacy bodies. Two Trusted Third Parties linked the databases via a deterministic matching procedure using multiple encrypted social security numbers.

**Conclusion:**

In this article we describe how various routinely collected population-level databases and disease registries can be accessed and linked to study patterns in the use, quality and costs of end-of-life care in the full population and in specific diagnostic groups.

**Electronic supplementary material:**

The online version of this article (doi:10.1186/s12904-016-0159-7) contains supplementary material, which is available to authorized users.

## Background

It has been argued that there is a particular challenge for end-of-life care research to develop a public health approach [1] which would include, among other things, the need for a focus on total populations instead of individuals at risk or those receiving a certain health care service. This means that many, often ‘hidden’, publics also need to be studied [1, 2]. End-of-life care research indeed often suffers from selection bias, recall bias and non-response bias [3–5] and difficult-to-reach populations tend to be under-represented due to ethical and practical considerations [6].

Administrative data can provide a major opportunity in this respect. They allow not only the monitoring of usage, quality and costs of end-of-life care on a population level [7], but also identifying populations dying of or dying with a specific disease such as cancer, chronic obstructive pulmonary disease (COPD) or Alzheimer’s disease in order to evaluate patterns of end-of-life care within and across different trajectories of dying [8]. Many healthcare institutions generate, store and exchange large amounts of individual patient data [9]. Increasing digitalization in recent years has further facilitated and improved this process [10]. Although big data serve administrative purposes particularly (e.g. billing, tracking of health care reimbursement) they can provide useful research material from a public health perspective [11]. They often have a well-defined population and include subgroups or difficult-to-reach populations [6]. Because administrative data registrations are usually standardized and continuously collected they enable trend analyses and longitudinal studies. Moreover, since the data have already been collected, they are relatively inexpensive when compared with original data collections [2, 12]. The expanding availability and quality of data input make them increasingly interesting to use in health research. Although full-population databases have been used to study end-of-life care since the late nineties (e.g. in Australia [13] and Canada [14]), the use of such data in end-of-life care research is still under-explored.

End-of-life care researchers may face several challenges when using administrative data. Administrative data are, for instance, not specifically designed for research purposes and therefore not directly usable for the evaluation of quality of care or quality of dying. They are not structured in readily available variables for analysis and may often lack the essential disease-specific or relevant socio-demographic information needed in end-of-life care research. Additionally, healthcare data, socio-demographic data, socio-economic data and clinical data gathered on every citizen are stored in separate databases that are owned and handled by different organizations. Also, data security and confidentiality must be publicly guaranteed when using administrative databases for healthcare research. The challenge is thus to collect, link, integrate, store and process them so that they provide a useful input for end-of-life care research.

Using the case of Belgium, we describe how several full-population data sources can be accessed, linked, handled and stored in order to obtain a rich database for evaluating the use, quality and costs of end-of-life care. Our research questions are: (1) what data and databases are available that provide information about end-of-life care, (2) what are the procedures to obtain/use these data, (3) what is needed to integrate separate databases, and (4) what variables are available in these databases to study use, quality and costs of end-of-life care.

## Methods

To address our research aims we systematically collected the necessary information in four phases:

1. First, we had to identify what databases provide information on the health care use, quality and costs near the end of life. We aimed to retrieve healthcare use data from all decedents for the two years prior to their death. A group of end-of-life researchers and health economics experts explored what data are available on healthcare and medication use that additionally (1) allow identification of people dying with or from cancer, Alzheimer’s disease or COPD; and (2) provide relevant socio-economic and demographic information that is known from literature to influence end-of-life care patterns. Health claims data were used as the starting point as they provide critical data about patterns in formal care and medication prescription at the end of life. Other administrative databases and disease registries were explored to supplement the health claims database.
2. Once the databases and the data handling organizations were identified, the associated access procedures and permissions as well as linking possibilities were explored.
3. To complete the linking procedure, technical aspects and privacy protection measures were determined, explored and followed.
4. Finally, we composed an overview of available variables through this process. We examined how they can be used to study use, quality and costs of end-of-life care.

## Results

### Identification and selection of databases

A total of seven population-level databases handled by three different organizations were identified as providing the necessary information (Table 1).

**Table 1:** Overview of population-level databases identified as relevant for end-of-life care research

Database administrators	Database name	Population	Information provided in database
Inter Mutualistic Agency (IMA)	Population Database	Every Belgian citizen who is a member of one of the seven (compulsory) Belgian sickness funds, information in Population Database is updated twice each year from 2002 onwards	Socio-demographic characteristics (age, sex, date of death, place of residence, family composition, use of supportive measures)
	Pharmanet Database		Medication supply characteristics (substance, quantity, prescriber, expenses, refunds, delivery date)
	Medical Claims Database		Health and medical care use characteristics (quantity of use, reimbursement, supplier, supplier institution, length of treatment)
Belgian Cancer Registry	Cancer registry	Every new cancer diagnosis of Belgian residents, registered by oncological care programs and laboratories for anatomic pathology	Diagnostic characteristics (date of diagnosis, type of cancer, TNM gradation)
Statistics Belgium	Death certificate database	Every Belgian decedent with a registered death certificate	Direct and indirect causes of death (in ICD-10 codes), socio-demographics about the deceased, place of death
	Demographic dataset	Every Belgian citizen	Nationality group, household composition
	Socio-economic survey (SES) 2001 and Census 2011	Every Belgian citizen, information gathered from multiple external administrative databases using social security number (Census 2011)	Highest attained education level, occupation, housing comfort
	IPCAL dataset	Every Belgian citizen	Net income by category
Identified but not used in our research			
Belgian Ministry of Health	Minimal Hospital Dataset	Every hospital admission in non-psychiatric general hospitals	Medical, nursery and personnel data for in-hospital care

The Inter Mutualistic Agency (IMA) manages the databases that included all reimbursement data of health care consumption from all seven healthcare insurers. Since health insurance with one of these insurers is legally mandatory in Belgium, reimbursement data of all legal residents are available in the IMA database. Moreover, thorough quality procedures result in reliable usability of the database for healthcare research. The IMA manages three databases: (1) a population database containing socio-demographic data of all insured persons; (2) a health care database containing health care use and costs data of both ambulatory and hospital care and (3) a pharmaceutical database containing medication prescription and costs data. The databases thus provide information on an individual level across the entire Belgian population. The IMA databases contain no information regarding medical diagnoses or any disease specific information.

The Belgian Cancer Registry was identified as a database to identify people who died with cancer. All Belgian oncological care programmes of hospitals and laboratories for anatomic pathology are legally bound to register each new cancer diagnosis with the cancer registry. The latter manages a database with diagnostic information on all incidences of cancer i.e. date of diagnosis, type of cancer and TNM (tumour node metastasis) classification of malignant tumours [15].

However, the cancer registry data does not make it possible to distinguish between those who died ‘from’ cancer and those who died ‘with’ cancer. Additionally, since no similar registries were available to identify those who died with or from Alzheimer’s disease and COPD we identified the death certificate data as a necessary additional database. Death certificate data in Belgium are collected by three administrations (corresponding to the three semi-autonomous regions in the country, i.e. Brussels, Flanders and Wallonia) and are integrated by Statistics Belgium into one national database for cause of death statistics. This database provides the causes of death and associated causes of death (coded in ICD-10 [10th revision of the International Statistical Classification of Diseases and Related Health Problems] codes) for all decedents.

Statistics Belgium also manages the national demographic database, derived from the population register [16] and containing for example the household composition of every citizen and data from the Socio-Economic Survey 2001 and Census 2011, nationwide full population surveys based on the tradition of population count [17]. The database contains information about the highest educational level attained, the last held occupation (as a measure of socio-economic position) and housing characteristics, which are all socio-economic factors that have been identified in previous studies as affecting end-of-life care patterns [18–20]. Finally a database containing fiscal data (i.e. net taxable household income), also managed by Statistics Belgium, was identified as providing additional socio-economic variables of influence on end-of-life care patterns.

For more specific clinical data, the Minimal Hospital dataset, providing clinical information associated with hospitalizations, was looked at for possible inclusion. This dataset has high quality data and provides diagnostic information (in ICD-codes), which allows for a more exact clinical description of the study population. It is however limited to in-hospital data, limiting the study population. Additionally, clinical information can be abstracted from health care claims data using specific algorithms. Obtaining cause of death information and using healthcare claims data makes up for the lack of clinical data. Therefore inclusion of the Minimal Hospital dataset was found to be unnecessary.

The combination of identified databases would provide information on formal health care and medication prescription, causes of death, main diagnosis (through the cancer diagnostic information of the cancer registry and algorithmic estimation methods in the IMA databases), and various relevant socio-demographic and socio-economic information.

### Access procedures

Two types of approval were needed for every database: (1) internal approval from database administrator organizations and (2) approval from the relevant Belgian Privacy Commission bodies.

1. To obtain access to the IMA and cancer registry databases several steps are required. First, a declaration of interest needs to be set up between researchers and IMA and cancer registry programme managers. Research goals, databases, variables and linking possibilities (see Data linkage procedure) need to be discussed. After IMA and cancer registry programme managers agree on cooperation, the research project (research goals and requested data) is presented to IMA and cancer registry directory boards for approval.
  To obtain access to the databases administered by Statistics Belgium, no formal approval of the directory board is required, since Statistics Belgium is legally committed to providing data for research. Based on the requested data, variables and linking possibilities (see *Data linkage procedure*) the statisticians of Statistics Belgium deliver non-binding advice. Data requests should be filed directly to the Privacy Committee.
  All involved partners then discuss the final selection of data and variables and initiate preparations for the linking procedure. The linking of the databases is a main issue for approval by the involved Privacy Commission bodies.
2. We needed the approval of two separate national sectoral committees for privacy protection for access to the various databases and the database integrating all databases: the ‘Sectoral Committee of Social Security and Health, Section Health’ and the ‘Statistical Supervisory Committee’. Both are subcommittees of the Belgian Commission for the Protection of Privacy. The former is responsible for privacy protection of health care data (IMA and cancer registry databases), the latter for privacy of national statistical data (Statistics Belgium databases). The application to the Sectoral Committee of Social Security and Health, Section Health consists of two phases. Phase 1 is the submission of the application and a first assessment by the committee in a plenary meeting. The primary investigator of the study presents the research goals and data linkage procedure to the committee at this plenary meeting. In our application special attention was given to the selection of different variables to receive data with sufficient detail for analysis, but at the same time reducing the risk of re-identification of deceased individuals and their families in order to preserve privacy. Changes in the linking procedure and storage on a separate server were requested (see section on data linkage for more details). Phase 2 is the formal approval during a second plenary meeting of the committee, after having received additional information from the applicant. In our case, the committee requested an additional risk analysis to ensure privacy of the included individuals, which was not requested in the first phase. Formal approval was granted only after a third plenary meeting. The full process took six months from application to formal approval.
  The Statistical Supervisory Committee application procedure consists of one phase in which the application is assessed and discussed on a plenary meeting. Formal approval was granted after the first meeting.

### Data linkage procedure

All eligible databases needed to be linked into one integrated database for analysis; a common unique identifier (i.e. social security number) made deterministic linking possible. Although the death certificate database does not contain this unique identifier, Statistics Belgium performed a linkage between the death certificate database and the national registry database based on date of birth, sex, and municipality of residence in order to include this unique identifier as a variable. Unique linkage was possible for 98.4 % of deaths.^1^

**Fig. 1:**
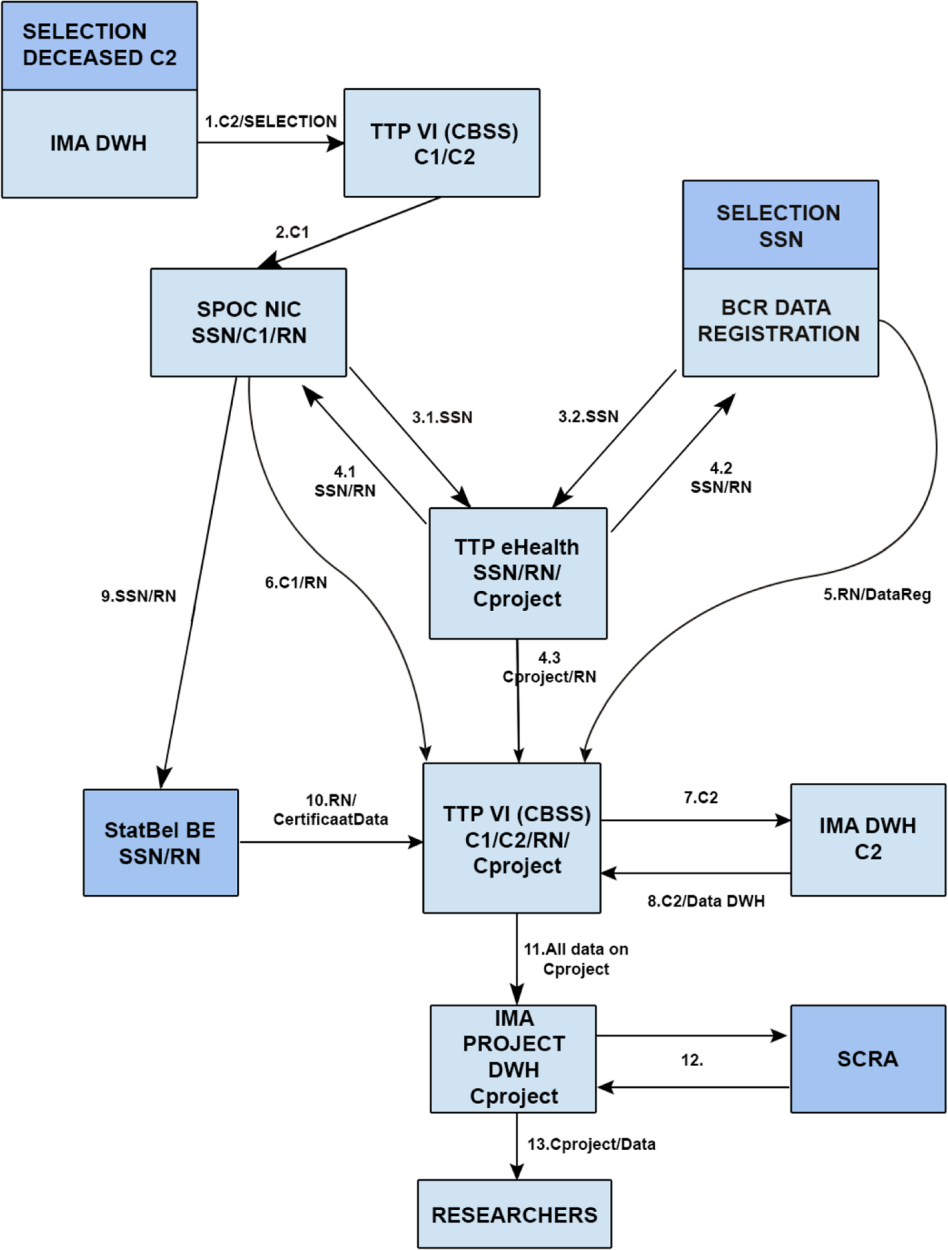
Step-by-step overview of linkage procedure. IMA DWH: InterMutualistic Agency Data Warehouse; TTP VI CBSS: Trusted Third Party Crossroads Bank for Social Security; SPOC NIC: Single Point of Contact National InterMutualistic College; BCR: Belgian Cancer Registry; TTP eHealth: Trusted Third Party eHealth; StatBel BE: Statistics Belgium; SCRA: Small Cells Risk Analysis; SSN: Social Security Number; C1/C2: coding 1/2; Explanatory note: The linkage procedure consisted of 13 steps (cf. arrows Fig. 1). Step 1: All cases from Belgian decedents since January 1, 2010 are selected in the IMA databases with their specific identifier coded (C2). These are then decoded (C1) by the TTP VI (CBSS). Step 2: The security officer of the National InterMutualistic College decodes the identifiers (C1) into actual social security numbers. Step 3.1: The IMA subset of social security numbers is sent by secure means to the separate TTP eHealth. Step 3.2: TTP eHealth receives the social security numbers from all cases in the Cancer Registry selected for the study (decedents since January 1, 2010). Step 4.1 and 4.2: To avoid any party from having access to both the sensitive data and the social security numbers, the established principle of random transport numbers (RN) is used. TTP eHealth assigned these RNs for the selected cases from IMA (4.1) and BCR (4.2) and provides these RNs to both data agencies in order to transmit the sensitive data safely to the TTP VI (CBSS). Step 4.3: TTP eHealth recodes the social security numbers into a final code that can be made available to the researchers (Cproject). These are sent, with the RNs as a cross reference coding, to the TTP VI (CBSS). Step 5: The selected cases and the corresponding requested data from the Cancer Registry are securely transmitted to the TTP VI (CBSS). Step 6: The selected IMA cases (but not yet the corresponding requested data) are securely transmitted to the TTP VI (CBSS). Step 7: The selected cases are transferred to the IMA datawarehouse (based on C2) so as to allow the selection of all data corresponding to these cases. Step 8: The selected cases and the corresponding requested data from IMA are securely transmitted to the TTP VI (CBSS). Step 9: The social security numbers and corresponding RNs are transferred safely to Statistics Belgium in order to allow selection of the correct cases. A social security number has already been attributed by Statistics Belgium to every case in the death certificate data (which do not contain the social security numbers) based on a deterministic linkage between the death certificate database and the national registry database based on date of birth, sex, and municipality of residence. Step 10: Statistics Belgium sends the requested data from the selected cases to TTP VI (CBSS) who links these with the data from IMA and Cancer Registry using the RNs. Step 11: The TTP VI (CBSS) recodes all data one final time based on the Cproject coding. Step 12: A small cells risk analysis is performed to minimize the risk of re-identification based on a combination of variables. Step 13: The complete linked database is stored on a separate IMA data server, which is only accessible to the researchers through a Virtual Private Network (VPN) connection with secure token

For privacy reasons, Trusted Third Parties (TTPs) ‘eHealth’ and ‘Crossroads Bank for Social Security (CBSS)’ were responsible for the simple deterministic one-to-one record linkage of the IMA, cancer registry and Statistics Belgium databases. The linkage procedure (Fig. 1) consisted of 13 steps of data-coding or decoding and data transfers needed to ensure that none of the involved parties would have access to both the sensitive data and the social security numbers or to their own databases enriched with data from one of the other parties. Only the researchers have access to the complete linked database without unique identifiers using a Virtual Private Network (VPN) connection with secure token.

Linkage of all data for deaths in 2012 (including health care information about the two years prior to death) were completed in a first phase of the project. In a second phase all data for all deaths 2010-20xx are linked, where data from subsequent years will be added upon availability. A major consideration in the decision to adopt this phased approach is the size of the linked database. The linked database (deaths in one year) will be used for the initial analysis, after which a selection of variables and/or information can be made. Variables with too many missing data or variables that are inaccurate can be dropped. Additionally, the initial analysis will inform on what health care interventions or medications are suitable for further analyses. Finally, based on this first analysis phase, detailed information can be aggregated. The second and third delivery will therefore include more cases with more condensed information per case.

Since all databases depend on submission by individual organizations or institutions, a two-year delay is common. Linkage can only be initiated after all data are complete.

### Available information and data handling

Variables selected in this study include data on health care use, prescribed medication, demographics, socio-economics and use of special reimbursement rules. A complete list of variables can be found in Additional file 1: Table S1.

Several steps were necessary to make the data analysis-ready:

1. In the IMA databases, health care and medication data are coded as nomenclature and Anatomical Therapeutic Chemical Classification (ATC) codes. In order to answer research questions, nomenclature numbers had to be interpreted and possibly aggregated by the researchers into meaningful categories.
2. Due to privacy concerns, no raw dates (e.g. date of birth, prescription date) were provided by database administrators. Dates of medication delivery or health service provision were therefore transformed into a number of days before death. Combinations of these recoded dates and nomenclature or ATC codes are used to determine whether certain interventions occur within a certain time period before death.
3. Since no data were provided on diagnosis in the current set of linked databases (only causes of death are available), algorithms were used to abstract diagnostic information from health care and medication prescription data. Algorithms were developed to identify people with COPD or Alzheimer’s disease, based on treatments and medication received. The algorithms were developed using existing evidence [21–23] and were validated by medical experts and medical data experts from the IMA. They were then applied by the IMA, prior to the linking procedure, because data were used that were not available to the researchers; data provided to the researchers were limited to two years prior to death, while data used for identification of patients with Alzheimer’s disease went back to six years before death. Combinations of the algorithmic identification of diagnosis and the causes of death (including the associated causes) can be used to identify relevant disease groups in the analyses.

## Discussion

### Summary of main results

In linking information from seven different datasets we managed to obtain a database that can provide information about patterns in the use, quality and costs of end of life care at the level of the full population and their associations with various clinical, socio-demographic, socio-economic and environmental factors. The process of obtaining this involved detailed identification of databases fitting the study aims, negotiation with and formal approval of three database administrators, three supervisory bodies and two national privacy commissions and eventual linking of all databases through two Trusted Third Parties (TTPs) using multiple encrypted social security numbers.

We believe that the described process can be particularly helpful to researchers in other countries in compiling similar population-level databases on end-of-life care. A number of considerations (limitations, strengths and opportunities) and recommendations can be made based on our experience.

### Limitations of our study

Our study involved a systematic and thorough exploration of how several databases providing information on end-of-life care can be accessed, handled and linked into an integrated and enriched database. However, an important limitation is that linkage with information on patient-related outcomes of healthcare services, such as specific Patient Reported Outcome Measures (PROMs) was not explored in our study. Even though PROMs are important indicators to evaluate whether increased healthcare expenditure results in better health outcomes, their inclusion in a population-level database is only meaningful if there is sufficient standardization in the measurement methodology. In Belgium, a common coding system for PROMs is lacking and would be time-consuming to perform. [24] Future efforts could be made to include PROMs at a population level.

### Opportunities of the collected database

Our efforts resulted in a population-level database with detailed information about formal end-of-life care, the costs of care and demographic, socio-economic and diagnostic information on decedents. The opportunities provided by such a database to study use, quality and costs of end-of-life care are considerable. The main overall advantage is that data are population-level and therefore not subject to sample bias such as in surveys or medical records studies of selected groups of patients. Compared to primary data collection, using linked routinely collected databases as in our case is less expensive and less time-consuming. In the end-of-life care context specifically, primary data collection can be burdensome for patients and caregivers. Furthermore, in routinely collected databases, high-quality data are available on the spot, although they are not deliberately collected for research aims.

Although the linked database does not include certain types of information that are important in evaluating quality of care, such as patient-specific preferences of care, psycho-social information, patient or family reported outcomes and experiences or information about pain and symptom management or communication aspects [25], the full-population data have the potential to provide robust and population-level measures of the quality of end-of-life care using specific claims-based quality indicators. These quality indicators, e.g. mapping inappropriate end-of-life care, have been used in various studies as measures for the quality of end-of-life care [26]. If preceded by an adequate validation process, they can provide a detailed image of the quality of end-of-life care by regions or health care providers [26]. The linked database also contains data on all direct medical costs and reimbursed service and medication use, which offers opportunities to study direct medical costs and patterns in the use of specific end-of-life care for full populations. Policy measures that support palliative care include financial compensation directed towards the patient (e.g. monthly lump sum to cover additional costs for palliative home patients). Using this database, patterns in the uptake of these measures can be mapped and compared between population or pathology groups. As the linked database contains individual data, these data can be aggregated on multiple levels, which makes longitudinal, disease-, treatment- or provider-specific analyses possible. As a result, it is also possible to evaluate the influence of certain policy measures and governmental support programmes. Without the rich population-level data we collected it would be impossible to answer these example research questions without facing major issues of reliability, generalizability, feasibility and costs.

Data allow us to follow back the treatment history and costs of those treatments up to two years before death. Although a shorter period before death may be sufficient to study several aspects of end-of-life care in specific disease groups, for other (particularly non-cancer) longer time periods are warranted. The decision to request all health care and medication data up to two years before death (irrespective of when the diagnosis was made) was also made for practical reasons as going further back would substantially increase storage and analysis requirements.

### Limitations of the collected database

A limitation of these types of routinely collected population-level data is that services not covered by insurers are not included. Researchers from other countries that wish to compile a similar database need to remember that what is not covered by insurance (and hence not found in the data) may be country- or even region-specific. In Belgium, data are relatively complete, for health care services in the hospital, nursing homes and at home. Nevertheless use of certain services cannot be identified because there is no individual reimbursement (e.g. mobile hospital palliative care teams) or such reimbursement is not regulated or generalized (e.g. consultations of a psychologist). Secondly, total out-of-pocket spending is not available in the integrated database. This results in an overall underestimation of the total cost of end-of-life care. Nevertheless, administrative data are an essential source of information for studies on the financial burden of end-of-life care for the health care budget and are valuable for policymakers in informing their decisions on health care policy [8].

### Considerations and actions for researchers considering similar database constructions (Table 2)

**Table 2:** Considerations for researchers planning to link databases

Topics	Considerations
Exploring relevant databases	Are my research questions clear and well-defined? What data are needed to answer them?
	What is/are my study population(s)? What data are needed to identify it?
	What database(s) contains the core data and could thus be selected as a starting point?
	When a starting database is chosen, what data are lacking to fully address the research questions? Where can we find them?
	How can we establish contact with the database administrators of the databases? Obtain principal approval from all administrators (e.g. by presenting the study to the board of directors)
	What is the cost associated with each database?
Variable selection	What specific variables do we need from the selected databases to answer our research questions?
	Are the variables we want available and linkable between the different databases?
	Does the preferred selection of variables complicate the linking procedure considerably? Balance the gain in information with the increase in complexity and time.
	What is the required level of detail for each variable? Balance the preferred level with what is allowed in terms of data protection (e.g. through small cells risk analysis to determine risk of re-identification based on a combination of variables)
	Do we have sufficient storage capacity and analysis hardware to store and analyze all the data we want?
Access procedures	What ethical and privacy procedures need to be followed to link and access the selected database?
	What technical procedures need to be followed to link and access the selected databases?
Infrastructure	How will data be stored safely? Is infrastructure provided by researchers or by database administrators? What is the cost for this infrastructure?
	How will data be protected? Physical and digital protection need to be guaranteed.
	How can data be accessed in a safe and easy way? What hardware and software do we need to access and analyze the requested data?

The linkage process is crucial in obtaining a useful population-level database. It enables the enriching of the population-level data on formal end-of-life care and the costs of that care with putative demographic, socio-economic and diagnostic information for the study of end-of-life care patterns. This allows the development of explanatory models and the provision of public health information to policy makers, for example on social differences and differences between pathology groups. It can support discussions on the organization of the health care system, based for instance on possible existing inequities.

The flip side is that the linking can create additional difficulties in the process of obtaining the data. While deterministic linking is relatively easy to complete on a technical level (even without identical unique identifiers a deterministic linking is possible based on a combination of variables), the main challenges for researchers lie in the fact that 1) several separate organizations have to be convinced to cooperate and 2) special attention needs to be given to privacy-related issues.

Databases across health and social care may not always contain a unique identifier variable, or not always contain accurate and fully available information that allows identifying unique persons. In such cases where the possibility to perform deterministic linkage is limited the method of probabilistic linking can present a solution. In this approach the likelihood of a correct linking is calculated and a linking is done when the likelihood is sufficiently large [27]. Several tools have been developed to perform this probabilistic linking [28]. Nevertheless, a lack of accurate and fully available personal identifiable information constrains a probabilistic linking method.

A final consideration for researchers who wish to have access to similar data in their country is that establishing and maintaining good relationships with database administrators is crucial. Gaining access to administrative data is an iterative process that requires a lot of preparatory work. Database administrators are the researchers’ access points to the data and have all the information about internal procedures. Strict procedures need to be followed, in close cooperation with database administrators. We were able to arrange an updated dataset where data from subsequent years will be added upon availability in the same approval and agreement, which limits the time of going through all necessary permissions each time an updated dataset is needed. Since administrative data are often not gathered with the intention of research, or only for internal use, the process of making the data analysis-ready can take time. Researchers must adapt to how data are registered and stored, before they can effectively use them for research.

## Conclusion

Linking and accessing various routinely collected population-level databases involves challenges but offers substantial opportunities to study patterns in the use, quality and costs of end-of-life care both in the full population and for specific diagnostic groups. This study has identified that it is possible to combine data from different databases in order to obtain a rich database for such analysis, including information about all reimbursed care and medication as well as disease, demographic, socio-economic and environmental information. While some aspects may be specific to the Belgian context, our study has a much broader application as most developed countries collect similar population-level databases. The process described in our study can be a helpful aid for researchers in these countries to compile similar data and eventually develop an international comparative end-of-life care research agenda using administrative health care data.

## Additional file

Additional file 1: Table S1. Complete list of variables in the linked dataset (IMA – Statistics Belgium – BCR). (DOCX 22 kb)

## Abbreviations

ATC: Anatomical therapeutic chemical classification
CBSS: Crossroads bank for social security
COPD: Chronic obstructive pulmonary disease
ICD-10: International statistical classification of diseases and related health problems
IMA: Inter mutualistic agency
PROMS: Patient reported outcome measures
TNM: Tumour node metastasis
TTP: Trusted third party
VPN: Virtual private network.

## Footnotes

1 Between 1998 and 2013, 1,662,550 of 1,689,740 deaths were matched (98.39 %).
