# Supplementary material for: Using linked administrative and disease-specific databases to study end-of-life care on a population level
Source: BMC Palliat Care. 2016 Oct 18;15:86. doi: 10.1186/s12904-016-0159-7 (PMC5069861; doi:10.1186/s12904-016-0159-7)
Supplement: Additional file 1: Table S1. — Complete list of variables in the linked dataset (IMA – Statistics Belgium – BCR). (DOCX 22 kb) [file 12904_2016_159_MOESM1_ESM.docx]

*Additional file 1: Table S1: Complete list of variables in the linked dataset (IMA – Statistics Belgium – BCR)*

| **Flag** | **Variable** | **Description** |
| --- | --- | --- |
| **Statistics Belgium: Death certificates database** | | |
|  | Immediate cause of death | The primary disease/cause prior to death, + up to 3 underlying causes. |
|  | Associated causes of death | Up to 3 factors that indirectly contributed to death. |
| **Statistics Belgium: data based on demographic datasets** | | |
|  | natgr | Nationality-group: indicates to which of 16 most common nationalities in Belgium someone belongs. Less common nationalities are aggregated. (e.g. EU-other, Europe-other, other) |
|  | liprohht | LIPRO-household type: e.g. single parent, married with/without children, … |
| **Statistics Belgium : Socio-economic survey 2001 and census 2011** | | |
|  | q9a/EDU | Highest level of education. |
|  | q16a_m/SIE | Main profession. |
| **Statistics Belgium: Composite variables socio-economic survey 2001** | | |
|  | comf | Housing comfort level, based on number of types of different rooms (e.g. kitchen, bathroom…) and heating system. |
| **Statistics Belgium: IPCAL dataset** | | |
|  | Net income | Net income in the year prior to death. Provided relative to the entire population, not in absolute numbers. |
| **IMA: Population database** | | |
| ANON_BASE | Recoded PP0010 and SS00010 | Unique identification of the rightful claimant (coded) |
|  | PP0015 | Age (based on year of birth) |
|  | AGE05_CAT | Age of the rightful claimant in categories of 5 years, calculated on December 31 of the reference year. |
|  | PP0020 | Sex |
| Care region (based on NIS code) | Recoded PP0025 | Care region on the hospital level (in Flanders) |
| PROVINCE/DISTRICT | Based on PP0025 | Official place of residence at time of death |
| URB_CAT | Based on PP0025 | Degree of urbanisation of the place of residence |
|  | PP0030 | Social status (e.g. working, retired, …) |
|  | PP0040 (A, B, C) | Year of death |
|  | PP1010 | Indicates whether the claimant received enhanced reimbursement. |
|  | PP2001 | Indicates whether the claimant received a forfeit class B nursing care. |
|  | PP2002 | Indicates whether the claimant received a forfeit class C for nursing care. |
|  | PP2003 | Indicates whether the claimant received a forfeit class E for physiotherapy. |
|  | PP2005 | Indicates whether the claimant received the allowance for the integration of disabled persons (category III, IV, V). |
|  | PP2006 | Indicates whether the claimant received the allowance for assistance to the elderly (category III, IV, V). |
|  | PP2007 | Indicates whether the claimant received a payment for assistance of third person carers. |
|  | PP2008 | Indicates whether the claimant received an increased allowance for help from third parties. (based on degree of disability) |
|  | PP2009 | Indicates whether the claimant received a lump sum benefit for ‘assistance to others’. |
|  | PP2010 | Indicates whether the claimant was hospitalised at least 120 days during the last 2 years. |
|  | PP2011 | Indicates whether the claimant was hospitalised at least 6 times during the last 2 years. |
|  | PP3004 | Reimbursement category of the family. |
|  | PP3005 | Reimbursement category of the individual. |
|  | PP3006 | Date of the first claim entitled for maximum billing (provided in days prior to death). |
|  | PP3011 | Indicates whether the claimant received special allowances for disabled persons. |
|  | PP3014 | Indicates whether the claimant was entitled for maximum billing for the chronically diseased. |
|  | PP4002 | Number of days of unemployment due to disability. |
|  | PP4003 | Number of days of disability. |
|  | PP4004 | Evaluation of degree of functional status |
| CHRONICAL_YN |  | Indicates whether the claimant had at least one chronic illness in the last year and/or was entitled to an allowance for disabled persons. |
| IC_AVAIL_SA11 - IC_AVAIL_SA26 |  | Estimation of the availability of family and informal caregivers, based on age and social status of family members. |
| **IMA: Medical claims database** | | |
|  | SS00015 | Relative starting date of provision |
|  | SS00020 | Nomenclature code |
|  | SS00050 | Number of cases of provision |
|  | SS00055 | Number of days of provision |
|  | SS00060 | Amount of reimbursement |
|  | SS00065B | Caregivers’ qualifications |
|  | SS00070B | Prescribers’ qualifications |
|  | SS00075 | Identification of institution of the caregiver or prescriber (coded, not nominative) |
|  | SS00080 | Department code of the institution where care was provided |
|  | SS00085 | Place of care delivery (coded) |
|  | SS00105 | Number of institution which receives the payment |
|  | SS00110 | Date of hospitalisation |
|  | SS00115 | Date of hospital discharge |
| NEW_YN |  | Indication whether provided care is performed at weekends or at night |
| STAY_NR |  | Date of hospital admission |
| STAY_CAT |  | Type of hospital admission |
| ADMISSION, ADMISSION_YYYY |  | The first day that a stay is charged at a residence |
| DISCHARGE, DISCHARGE_YYYY |  | The last day that a stay is charged at a residence |
| LOS |  | Length of stay |
| LOS_YYYY |  | The calculated length of stay in a year |
|  | SS00120 | Invoice type |
|  | SS00125 | Date of last performance |
|  | SS00130 | Invoicing performance code |
|  | SS00135 | Pharmaceutical product code |
|  | SS00140 | Specification code of provision of care |
|  | SS00150 | Billed nomenclature code |
|  | SS00155 | Prescription date |
|  | SS00160 | Patient co-payment cost |
|  | SS00165 | Supplement |
|  | SS00170 | Code implant |
|  | SS00175 | Third party payer |
| THIRD_YN |  | Indicates whether a third party payer was involved |
| HOSP_ADM | SS00075, SS00085, SS00105 | Identification hospital admission |
| HOSP_TRANS | SS00075, SS00085, SS00135 | Identification hospital transfer(s) |
| **IMA: Pharmanet database** | | |
|  | SS00015 | Delivery date |
|  | SS00020 | Medication reimbursement category |
|  | SS00050 | Drug quantity |
|  | SS00060 | National Health and disability insurance contribution 1 |
|  | SS00070B | Profession type of prescriber |
|  | SS00075 | Type of long-term care |
|  | SS00135 | Product number |
|  | SS00155 | Date of prescription (in days before death) |
|  | SS00160 | Out-of-pocket cost |
|  | SS00165 | Reduced repayment amount / Contribution of pharmacists |
|  | SS00180 | Reduced insurance contribution |
|  | SS00195 | National health and disability insurance contribution 2 |
|  | SS00200 | Fee coding |
|  | SS00210 | Supplement |
| PHARMACIST_C, PHARMACIST_CAT |  | Pharmacist C is the coded unique identification number of the supplier of the performance.  Pharmacist_cat indicates the type of the supplier of the performance |
| UNIT |  | Indicates the unit to which the quantity is specified |
| procedure_AH_cat, procedure_group, procedure_detail, procedure_cat |  | Formats into categories, sub-categories and cost of the nomenclature code as they are determined by the actuary of the National Health Care insurer |
| Prescriber_c, prescriber_cat |  | Prescriber C is the coded unique identification of the prescriber's performance. PRESCRIBER_CAT indicates the type of the prescriber |
| atc_prod_l |  | The different levels of the ATC code |
| **BCR – Cancer Registry** | | |
|  | inc_death_mm | Number of complete months between incidence date and date of death |
|  | ICD10_new | Tumour localisation (ICD-10 code) |
